# Chocolate Classification by an Electronic Nose with Pressure Controlled Generated Stimulation

**DOI:** 10.3390/s16101745

**Published:** 2016-10-20

**Authors:** Luis F. Valdez, Juan Manuel Gutiérrez

**Affiliations:** Bioelectronics Section, Department of Electrical Engineering, CINVESTAV, Mexico City 07360, Mexico; fvaldez@cinvestav.mx

**Keywords:** E-noses, olfactometer, metal oxide gas sensors

## Abstract

In this work, we will analyze the response of a Metal Oxide Gas Sensor (MOGS) array to a flow controlled stimulus generated in a pressure controlled canister produced by a homemade olfactometer to build an E-nose. The built E-nose is capable of chocolate identification between the 26 analyzed chocolate bar samples and four features recognition (chocolate type, extra ingredient, sweetener and expiration date status). The data analysis tools used were Principal Components Analysis (PCA) and Artificial Neural Networks (ANNs). The chocolate identification E-nose average classification rate was of 81.3% with 0.99 accuracy (Acc), 0.86 precision (Prc), 0.84 sensitivity (Sen) and 0.99 specificity (Spe) for test. The chocolate feature recognition E-nose gives a classification rate of 85.36% with 0.96 Acc, 0.86 Prc, 0.85 Sen and 0.96 Spe. In addition, a preliminary sample aging analysis was made. The results prove the pressure controlled generated stimulus is reliable for this type of studies.

## 1. Introduction

Food analysis has always been important in order to characterize the properties of foods and their constituents. To obtain this information, it is common to use sophisticated procedures capable of providing a complete fingerprint on a wide variety of characteristics associated with the composition, structure, physicochemical properties, and sensory attributes [1]. In the last two decades, the technology of aroma-sensors has been developed and is now a competitive analytical tool even with the classical analytical chemistry methods such as gas chromatographic techniques. These novel analytical tools known as Electronics Noses (E-noses) can be successfully used as a rapid screening technique in aroma analysis [2]. An E-nose is an electronic device that tries to emulate the mammalian olfactory system process of detection, recording, memory search and recognition of odors. Its main component is an array of nonspecific chemical sensors [3].

Nowadays, we can find several works on the E-nose field, each one with different goals like food products evaluation [4,5,6,7], environment safety [8,9,10,11], disease diagnosis [12,13,14], etc. Making a review of the mentioned systems in the last decade, we can divide E-noses into three main blocks, each one presenting a few variations:

- The “odor sampling” unit is the one in charge of supplying a controlled “stimulus” to the sensor array. The controlled features can be different for each device, and the most widely used is flow control.
- The sensor array contains more than one gas sensor, which might have different gas sensors technology, gas chamber shapes and sensor distribution.
- The data processing unit is the one in charge of recording and recognition of the odors. It uses a combination of multiple techniques to extract the useful data from the sensors signals. Some of these techniques are Linear Discriminant Analysis (LDA), Principal Component Analysis (PCA), Artificial Neuronal Networks (ANNs), Support Vector Machines (SVM) and other artificial intelligence tools. This block implementation can be found on a variety of devices from personal computer to a digital signal processor.

The purpose of this work is to analyze the response of a Metal Oxide Gas Sensor (MOGS) array to a flow controlled stimulus generated in a pressure controlled canister using our portable olfactometer design [15] and build an E-nose. The selected odor source was chocolate because it is a commercialized product worldwide and one of the most popular snacks in the world. In addition, there are various products and, usually, its real contents or ingredient quality is unknown. There are some works related to the characterization and evaluation of chocolate, they use different techniques and procedures like colorimetry, panelist groups, high performance liquid chromatography (HPLC), High-sensitivity-Proton Transfer Reaction Mass Spectrometry (HS-PTR-MS), Fourier Transformed Infrared Spectroscopy (FT-IR), Gas Chromatography (GC) or Mass Spectrometry, among others [16,17,18,19,20,21]. These studies indicate chocolate can be characterized by its volatiles composition being a mix of alcohols, aldehydes, esters, ketones, furans, pyrans, pyrazines, pyridines, pyrroles, phenols, pyrenes and thiozoles [19]. This composition may be sensed by the MOGSs array to make an evaluation of their quality, freshness or even its packing/storage quality. Finally, we will evaluate the possibility to integrate our olfactometer design and the build E-nose in a portable “product odor evaluation platform” to characterize other products with ease.

## 2. Materials and Methods

### 2.1. Samples

The chocolate samples were taken from 26 commercial chocolate bars that share one or more characteristics between them (a complete description of the set is shown in Table S1). Considering the label description, the different chocolate bars were grouped as follows: first classification according to chocolate type (dark, soft dark and milk chocolate), followed by a second classification with 5 groups based in the extra ingredients (fruit, oils, etc.) added to the bars, a third classification based in the sweetener (with sugar and sugar free), and a fourth one based on the expiration date (spoiled and unspoiled). The samples subdivision is shown in Table 1. All chocolate bars were stored in separated sealed airtight bags at room temperature.

### 2.2. Olfactometer

A homemade laboratory olfactometer [15] was used to control the sensor array stimulation. The main function of an olfactometer is to produce a controlled olfactory stimulus. In our design, the stimulus is produced by controlling the pressure inside the odor canister (a 100 mL glass flask where the liquid or solid odorant samples are placed) and activating the output valve of the desired channel. The olfactometer can make mixes between 4 odor channels and a clean air channel. It can use the clean air channel to generate a carrier flow and dilute the odor stimulus if needed. 

**Figure 1:**
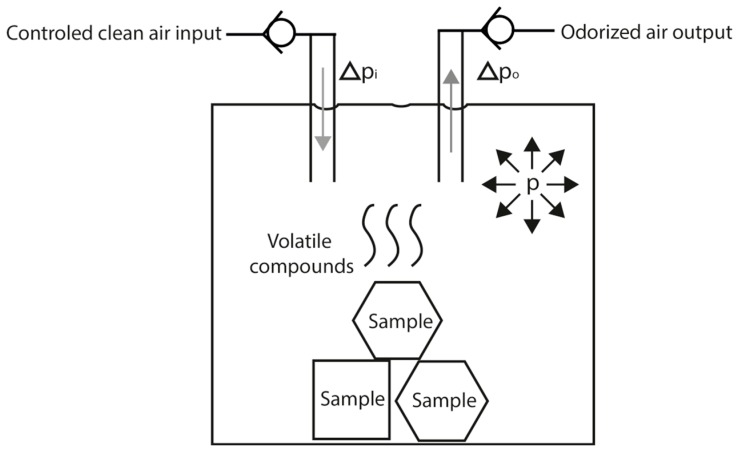
General odor canister dynamics.

Figure 1 illustrates the general dynamics of one channel when its output is activated. The pressure in the canister (p) is regulated by controlling the clean air input giving the needed increase of pressure (Δp_i_) to reach the desired pressure and after it is achieved it will only compensate the pressure decrease (Δp_o_) produced by the odorized air output. With the canister pressure controlled, the output flow will also be controlled. Considering all the experiments were conducted in laboratory-controlled environment (room temperature of 25 °C) and that the temperature of the canister is the same as the room temperature a regular volatilization of the volatile compounds in the sample can be achieved. This process will be ruled by the vapor pressure of the volatile compounds mixture and the selected system pressure, but we must consider that a steady concentration of the volatile compounds at the output will be obtained only if the composition of the mixture remains the same.

The test routine will be programed using its graphical interface and signal acquisition will be synchronized using the olfactometer digital outputs.

### 2.3. E-Nose Sensor Array

E-nose sensor array case design and sensors characteristics.The sensor array used was composed by 7 commercial MOGSs plus a temperature sensor. The gas sensors were placed inside of a 3D printed case made of polylactide (PLA) and the temperature sensor is located in the middle of the array as shown in Figure 2. O-rings were placed between the sensors and the case to avoid possible leaks. Finally, the olfactometer was connected to the sensor array via polytetrafluoroethylene (PTFE) tubing with 30 cm length and 1 mm of intern diameter.

### 2.4. Test Settings

**Figure 3:**
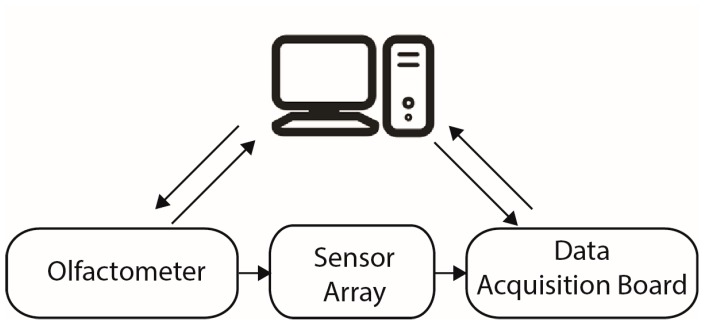
General setting of the system.

The test platform was built using the reported olfactometer [15] to control the stimulus, the sensors array, a data acquisition board (Measurement Computing, PMD-1608FS) and a personal computer as is shown in Figure 3. Two sensor arrays were constructed; one was used in experiment 1 and the other in experiment 2. 

A sensor array preparation routine was executed before the tests were started; it consists on the activation of the sensor array heaters and the carrier airflow (clean air channel at 250 mL/min) at least 72 h before the first sample was introduced to the olfactometer. This routine is necessary to achieve a steady response of the sensor array. The heater and carrier flow will not be turned off until the end of all the experiments.

Sensor array and synchronization signals were recorded with a sample rate of 10 Hz for experiment 1 and 1 Hz for experiment 2. The test sequence was:

1. Ten grams of a chocolate bar were placed inside a clean canister of the olfactometer (after the sample was taken the chocolate was stored in its airtight bag). For experiment 1, the canister was connected to the olfactometer right away. Instead, for experiment 2, a cap was placed and the sample was stored for 48 h before connecting it.
2. Two minutes of of 200 mL/min odor flow (generated by one of the olfactometer channels with the sample in and a canister pressure of 0.08 bar)
3. A cleaning sequence of 5 min pause, 2 min of 200 mL/min flow (generated by a second olfactometer channel with clean empty canister at 0.08 bars) and another 5 min pause.
4. Points 2 and 3 were repeated 40 times.
5. Wait at least 2 h and repeat from point 1 with the next sample.

Experiment 1 was conducted from 10 to 30 May and experiment 2 from 3 to 10 June. In addition, experiment 2 was only conducted for the first 10 chocolate bars. The reasons of the changes in experiment 2 will be explained later.

### 2.5. Signal Processing

After the acquisition of the signals, the 2-min sections of odor stimulation were segmented from the record. Each segment was centered by subtracting its mean value. Data analysis was made to evaluate the frequency and data content of the signals. After the analysis, it was decide to reduce the sample frequency of experiment 2 in order to reduce the data to process in the future when the E-nose is implemented in an embedded device.

The segmented data were organized in a data matrix; the experiment 1 data were subsampled using a moving average filter of 9 samples to 1 Hz making both experiment vectors the same length. The obtained data base size was 120 × 8 × 26 × 40 (samples, sensors, chocolate bars, and repetitions) for experiment 1 and 120 × 8 × 10 × 40 for experiment 2.

A preliminary PCA was made to evaluate the dispersion of the obtained data; gas sensor data was concatenated and all the tests were organized as rows for each experiment, giving a 1040 × 840 matrix to apply PCA for feature selection in experiment 1 and 400 × 840 for experiment 2. Afterwards, ANNs were used to build classification models to prove the acquired signals can be used to identify which chocolate is analyzed or identify some of its characteristics.

## 3. Results and Discussion

Recorded data were analyzed in order to identify differences between experiments or along them. Figure 4 shows the concatenated response of the gas sensors across the 40 test cycles in experiment 1 for chocolate G, where a decrease in the maximum value of the signal is observed from the first test to the last. We must consider sensor 1 shows a decreasing response so its minimum value is the one to be evaluated. The depletion of the volatile compounds in the samples or sample oxidation may be the responsible of this change so it can be associated with an aging process. 

To see if this process is also shown using an aged sample, the preparation protocol was modified by adding the 48 h storage of the samples before starting the test. In this way, we expect to capture an advanced stage of this aging process. The aging process is clearly visible by observing the results on Figure 5; there are no crests at the start of the test. However, the signals still show a decrease in the response of the sensors this may indicate the pressure controlled canister helps to have a regular depletion of the volatile compounds. Nevertheless, further tests are needed to identify if this measure can be used to evaluate this sample aging. If it is possible, it can be used for chocolates and similar products as a quality characteristic or to evaluate its packing/storage state.

The aging of the samples can only be attributed to the alteration or loss of their volatile compounds and not to the sensor aging drift. Aging drift was considered to be non-existent considering the whole experimental phase spans around one month and based on a study of long term stability of MOGS, the variations in the signal amplitude are around 2.5% per month [22].

**Figure 6:**
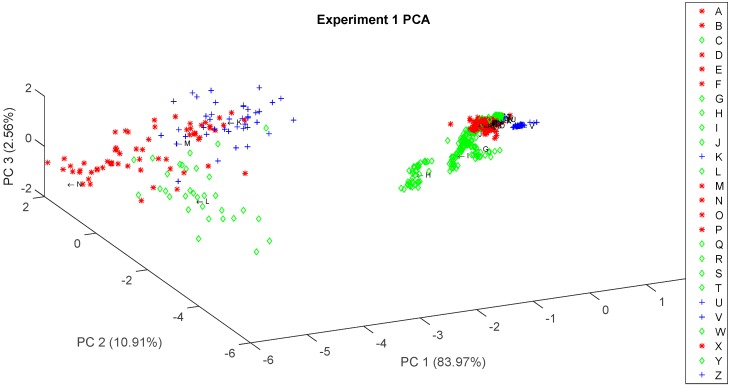
Experiment 1 PCA colored by chocolate type (Red, Dark; Green, soft dark; and Blue, milk). A to Z tags correspond to the chocolate samples listed in Table S1.

**Figure 7:**
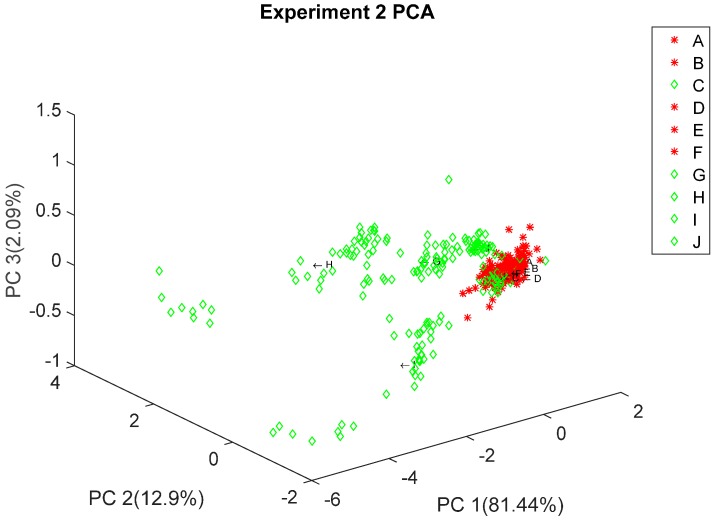
Experiment 2 PCA, colored by chocolate class (Red, dark; and Green, soft dark). A to J tags correspond to the chocolate samples listed in Table S1.

Once the pressure controlled generated stimulus proved to produce a stable response in the sensor array, a PCA analysis was done in order to build a preliminary recognition model, expecting to see some clustering produced by the variations in the volatiles composition of chocolate and brand-related processing as stated in [18]. Figure 6 and Figure 7 show Experiment 1 and Experiment 2 PCA results, respectively, where we can see some clustering based on chocolate type. Other PCA results can be consulted on the supplementary materials (Figures S1–S6) colored to see clustering associated to other characteristics such as extra ingredients, sweetener and expiration date.

**Figure 8:**
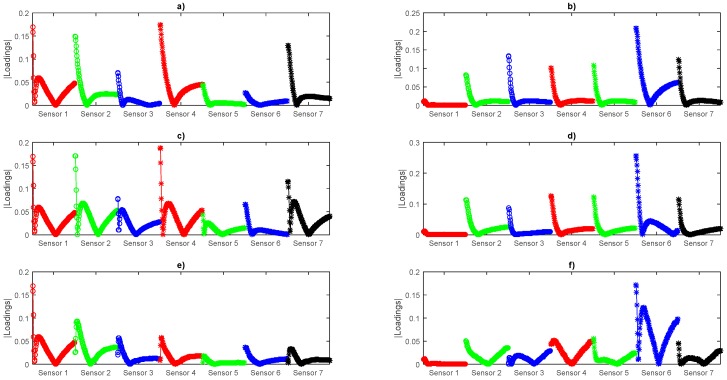
Experiment 1 and 2 loadings plot (X-axis corresponding to sensor array signals and Y-axis to the absolute value of the loadings). (**a**) Experiment 1 PC1; (**b**) Experiment 2 PC1; (**c**) Experiment 1 PC2; (**d**) Experiment 2 PC2; (**e**) Experiment 1 PC3 and (**f**) Experiment 2 PC3.

Besides the clustering, we can analyze the contribution of each sensor from the loadings of the first three components shown in Figure 8, where we can see in experiment 1 all the sensors contribute but in experiment 2 sensor 1 has almost no contribution to the Principal Components (PCs) indicating that it reacts almost in the same way for all the aged samples. PC4 to PC18 Loadings plots can be consulted in Supplementary Materials (Figures S7–S12). Nevertheless, we will make use of the whole array data according to the concept of an E-nose. 

The other objective of the PCA was focused on the dimensionality reduction of the input vector that feed the ANN. In this way, some of the PCs were selected, considering that the accumulative variance of the them was at least 95% [23]. The dimensionality reduction assures the constructed network will be easily implemented in hardware as part of an on-line identification device in the near future. Two different classifiers were made using Multilayer Perceptron Neural Networks (MLPs). The first one will identify which chocolate is being analyzed, so it will have one output neuron for each trained chocolate bar (experiment 1, 26 neurons; and experiment 2, 10 neurons). Several tests were made for the optimization of the network architecture and selection of the number of PCs to be used. The selected architectures based in its performance and dimensionalities were:

- Experiment 1: 15 × 10 × 26 with sigmoid tangent activation functions in the hidden and output layer. The classifier threshold was set from 0.8 to 1.
- Experiment 2: 10 × 6 × 10 with sigmoid tangent activation functions in the hidden and output layer. The classifier threshold was set from 0.8 to 1.

Training was conducted for each experiment with 75% of the data (30 cycles per sample) and tested with the remaining information (10 cycles per sample) using the Resilient Back Propagation (Rprop) training algorithm [24]. Training settings are shown on Table S2. Cross validation was made through K-fold validation algorithm with k = 4. 

The tests average results for 10 repetitions of training and validation after random selection of the training and test data is shown on Table 2 for experiment 1 and Table 3 for experiment 2. Experiment 1 classification rate is 93.2% for training and 84.2% for test. Experiment 2 classification rate is 94.6% for training and 79.7% for test.

We can highlight some trends in the results:

- In both experiments, the chocolates that contain fruit have a better classification rate than other chocolates, as expected from the PCA results.
- In experiment 2, from chocolates without fruit, the best classification is observed in Chocolate D. It has the peculiarity of being the only sugar free chocolate included in this test.
- In both experiments, Chocolates B and E have a low confusion rate between them despite being from the same brand. This may point the E-nose has the ability to differentiate between spoiled and fresh samples.

The second classifier used a 10-output codification; this network will try to classify the samples as shown on Table 1 to verify if it possible to identify these chocolate characteristics at the same time. The selected architecture was 30 × 10 × 10 with sigmoid tangent activation functions in the hidden and output layers because of its performance. Training was conducted as the previous classifier (75% training and 25% test). K-fold cross validation with k = 4 was performed and the test average results for 10 repetitions of training and validation after random selection of the training and test data is shown on Table 4 for experiment 1, giving an average classification rate of 85.36%. 

The results indicate:

- Chocolate samples can be correctly classified as dark, soft dark and milk chocolate regardless of its other ingredients. In addition, these ingredients can be identified.
- It is possible that the used sweetener modifies the volatile compounds and that is why sugar classifier have a good performance and the strawberry classifier has troubles classifying Q samples that have Splenda^®^ (Tate & Lyle, London, UK) as sweetener.
- Spoiled samples classifier has a bad performance but this can be caused by the lack of samples, so we propose to make another study with more spoiled samples in the future.

For experiment 2, the same classification was made but the outputs were reduced to eight based on the analyzed samples. The classifier architecture was 30 × 10 × 8 and the results are shown in Table 5. 

The results show good performance of the classifier but we must make a couple of notes:

- After the samples were aged, they could still be classified with similar performance.
- Spoiled sample detection was better in experiment 2. This can be explained if we consider the spoiled sample aging leads to the loss of the remaining volatile compounds or its accelerated decomposition.

The classifiers have a good performance considering the sensors used in the sensor array are one of the most simple and economic gas sensors available.

## 4. Conclusions

This study demonstrates that the use of commercial and nonspecific gas sensors to cocoa volatile compounds can be useful in the characterization of real chocolate samples without any pretreatment. The results prove that the pressure controlled generated stimulus is reliable for these types of studies, and the employed sensor array gives sufficient data for the proposed classification through a characteristic fingerprint of a mixture of volatile compounds present in each sample.

Connected to this, the differences between the performance of experiment 1 and experiment 2 classifiers indicate the possibility to identify an aging sample process that must be considered in future experiments. This preliminary finding suggests that the analysis of this aging process could be improved, not only by the measure of a wide range of volatile compounds (integrating other MOGSs to the current sensor array) but also using features related to the measuring conditions used. In addition, there is the alternative of evaluating sample aging itself, using features like the time or number of cycles that the maximum value of a signal takes to fall a certain percentage or others related to signal shape changes.

It is interesting to note that the integration between the olfactometer and the E-nose on a single device to obtain a “product odor evaluation platform” has shown good performance of discrimination and can be used to evaluate other products with ease in the near future.

## Figures and Tables

**Figure 2:** E-nose sensor array case design and sensors characteristics.

**Table:** 

**Sensor**	**Model**	**Brand**	**Main Detection**	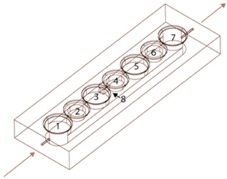
1	MQ-8	Winsen	Hydrogen	
2	MQ-9	Henan Hanwei	CO and combustible gases	
3	MQ-6	Winsen	LPG	
4	MQ-7	Parallax	CO	
5	MQ-5	Parallax	LPG, natural gas	
6	MQ-3	Parallax	Alcohol and Benzine	
7	MQ-2	Parallax	LPG, i-butane, propane	
8	LM 35	Texas Instruments	Temperature	

**Figure 4:** Gas sensors response to chocolate G stimulation across experiment 1. Bold numbers for the greater decrease.

**Table:** 

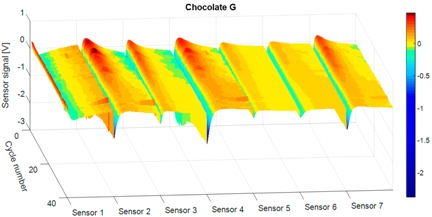	**Sensor**	**Decrease (%)**		
		**Max.**	**Min.**	**Amplitude**
	1	−2.82	**17.35**	4.39
	2	**69.72**	53.44	56.21
	3	**67.41**	51.23	55.65
	4	**61.55**	49.97	51.44
	5	**68.59**	48.49	52.77
	6	**64.71**	56.93	58.16
	7	**64.47**	45.04	48.58

**Figure 5:** Gas sensors response to chocolate G across experiment 2. Bold numbers for the greater decrease.

**Table:** 

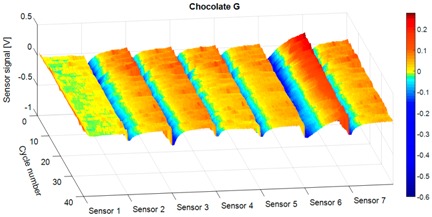	**Sensor**	**Decrease (%)**		
		**Max.**	**Min.**	**Amplitude**
	1	20.45	**2.93**	16.40
	2	**56.33**	71.84	68.73
	3	**32.73**	63.64	59.66
	4	**74.82**	79.50	78.80
	5	**49.81**	69.26	66.33
	6	**30.60**	51.04	44.44
	7	**47.47**	70.52	67.06

**Table 1:** Sample subdivision according to properties of interest.

Group/Characteristic	Total Number of Samples	Subgroup	Subgroup Tag	Number of Samples
Chocolate type	26	Dark chocolate	DK	10
		Soft darkchocolate	SD	12
		Milk chocolate	ML	4
Extra Ingredient	26	Coconut	CO	1
		Strawberry	ST	2
		Orange	OR	1
		Mint	MI	1
		Pistachio	PI	1
		None	-	20
Sweetener	26	Sugar	SU	21
		Sugar free	-	5
Expiration date status	26	Unspoiled	-	25
		Spoiled	SP	1

**Table 2:** Classification results of classifier 1 with experiment 1 data.

Classifier	Classification Rate (%)	Accuracy	Precision	Sensitivity	Specificity
A	82.50	0.99	0.80	0.83	0.99
B	82.25	0.98	0.78	0.82	0.99
C	65.75	0.97	0.65	0.66	0.99
D	78.75	0.98	0.77	0.79	0.99
E	47.75	0.97	0.68	0.48	0.99
F	45.50	0.97	0.66	0.46	0.99
G	95.75	1.00	0.95	0.96	1.00
H	97.25	1.00	0.94	0.97	1.00
I	98.25	1.00	0.97	0.98	1.00
J	97.50	1.00	0.96	0.98	1.00
K	93.50	1.00	0.95	0.94	1.00
L	85.50	0.99	0.91	0.86	1.00
M	85.50	0.99	0.89	0.86	1.00
N	90.75	0.99	0.93	0.91	1.00
O	71.75	0.98	0.79	0.72	0.99
P	75.25	0.98	0.81	0.75	0.99
Q	74.75	0.99	0.85	0.75	0.99
R	94.25	0.99	0.87	0.94	0.99
S	94.25	0.99	0.88	0.94	0.99
T	92.25	0.99	0.90	0.92	1.00
U	93.00	0.99	0.91	0.93	1.00
V	99.50	1.00	0.95	1.00	1.00
W	92.00	0.99	0.88	0.92	0.99
X	75.75	0.99	0.85	0.76	0.99
Y	90.75	0.99	0.83	0.91	0.99
Z	88.50	0.99	0.86	0.89	0.99
Average	84.2	0.99	0.86	0.84	0.99

**Table 3:** Classification results of classifier 1 with experiment 2 data.

Classified as: ^1^	A	B	C	D	E	F	G	H	I	J	Accuracy	Precision	Sensitivity	Specificity
A	**6.6**	1.7	0.1	0.1	0.0	0.0	0.0	0.2	0.0	0.0	0.98	0.76	0.66	0.98
B	1.5	**6.8**	0.1	0.0	0.1	0.0	0.0	0.0	0.0	0.0	0.98	0.81	0.68	0.98
C	0.0	0.0	**6.1**	0.1	0.7	0.9	0.0	0.0	0.0	0.0	0.98	0.77	0.61	0.98
D	0.1	0.1	0.2	**9.5**	0.2	0.0	0.0	0.1	0.1	0.0	0.99	0.94	0.95	0.99
E	0.0	0.0	0.8	0.1	**7.1**	0.4	0.0	0.1	0.1	0.0	0.99	0.86	0.71	0.99
F	0.0	0.0	1.1	0.0	0.7	**6.7**	0.1	0.1	0.0	0.0	0.98	0.77	0.67	0.99
G	0.0	0.0	0.1	0.0	0.0	0.1	**8.9**	0.2	0.0	0.3	0.99	0.92	0.89	0.99
H	0.0	0.1	0.1	0.0	0.1	0.1	0.4	**9.1**	0.1	0.1	0.99	0.92	0.91	0.99
I	0.1	0.0	0.1	0.0	0.0	0.1	0.0	0.1	**9.8**	0.0	1	0.97	0.98	1
J	0.0	0.0	0.0	0.0	0.0	0.0	0.3	0.1	0.2	**9.3**	0.99	0.93	0.93	0.99
Total classification rate						79.7%		Average			0.99	0.87	0.80	0.99

^1^ Bold numbers for correct classification.

**Table 4:** Test classification results of classifier 2 with experiment 1 data.

Classified as:	Classification Rate	Accuracy	Precision	Sensitivity	Specificity
DK	87.00	0.92	0.92	0.87	0.95
SDK	93.08	0.91	0.89	0.93	0.90
ML	88.56	0.96	0.87	0.89	0.98
CO	96.50	1.00	0.95	0.97	1.00
ST	73.38	0.97	0.89	0.73	0.99
OR	97.50	1.00	0.97	0.98	1.00
MI	95.25	1.00	0.94	0.95	1.00
PI	93.75	0.99	0.79	0.94	0.99
SU	93.61	0.92	0.96	0.94	0.86
SP	35.00	0.96	0.45	0.35	0.98
Average	85.36	0.96	0.86	0.85	0.96

**Table 5:** Test **c**lassification results of classifier 2 with experiment 2 data.

Classified as: ^1^	Chocolate Sample														
	A	B	C	D	E	F	G	H	I	J	C. Rate (%)	Acc.	Prc.	Sen.	Spe.
DK	**9.38**	**9.93**	0.13	**8.48**	**9.95**	**9.03**	0.03	0.00	0.08	0.00	93.50	0.97	1.00	0.94	1.00
SDK	0.08	0.00	**7.48**	0.08	0.00	0.15	**9.80**	**9.83**	**9.75**	**9.83**	93.35	0.96	0.99	0.93	0.99
CO	0.00	0.00	0.00	0.00	0.00	0.03	**8.03**	0.00	0.00	0.03	80.25	0.98	0.99	0.80	1.00
ST	0.00	0.00	0.03	0.00	0.00	0.00	0.03	**9.60**	0.00	0.00	96.00	1.00	0.99	0.96	1.00
OR	0.00	0.00	0.00	0.00	0.00	0.00	0.00	0.00	**9.60**	0.00	96.00	1.00	1.00	0.96	1.00
MI	0.00	0.00	0.00	0.00	0.00	0.00	0.00	0.00	0.08	**8.38**	83.75	0.98	0.99	0.84	1.00
SU	**9.23**	**9.78**	**8.95**	0.25	**7.70**	**9.63**	**9.95**	**9.95**	**9.98**	**10.00**	94.61	0.95	1.00	0.95	0.98
SP	0.00	0.00	0.00	0.03	**6.73**	2.8	0.00	0.00	0.01	0.00	67.25	0.96	0.88	0.67	0.99
									Average		88.09	0.98	0.98	0.88	0.99

^1^ Bold numbers for correct classification.

## Supplementary Materials

The following are available online at http://www.mdpi.com/1424-8220/16/10/1745/s1, Table S1: Chocolate samples description, Table S2: Training parameters for both classifiers, Table S3: Classifier 1, experiment 1 full test results, Table S4: Classifier 1, experiment 1 full training results, Table S5: Classifier 2, experiment 1 full test results, Table S6: Classifier 2, experiment 1 full training results, Figure S1: Experiment 1 PCA colored by extra ingredient, Figure S2: Experiment 2 PCA colored by extra ingredient, Figure S3: Experiment 1 PCA colored by Sweetener, Figure S4: Experiment 2 PCA colored by Sweetener, Figure S5: Experiment 1 PCA colored by expiration date, Figure S6: Experiment 1 PCA colored by expiration date, Figure S7: Experiment 1 PC1 to PC6 loadings plot, Figure S8: Experiment 1 PC7 to PC12 loadings plot, Figure S9: Experiment 1 PC13 to PC18 loadings plot, Figure S10: Experiment 2 PC1 to PC6 loadings plot, Figure S11: Experiment 2 PC7 to PC12 loadings plot, Figure S12: Experiment 2 PC13 to PC18 loadings plot, Test database.
