# Supplementary material for: Chocolate Classification by an Electronic Nose with Pressure Controlled Generated Stimulation
Source: Sensors (Basel). 2016 Oct 20;16(10):1745. doi: 10.3390/s16101745 (PMC5087530; doi:10.3390/s16101745)
Supplement: Supplementary file 1 [file sensors-16-01745-s001.pdf]

Supplementary Materials: Chocolate Classification by an Electronic Nose with Pressure Controlled Generated Stimulation

Luis F. Valdez and Juan Manuel Gutiérrez

Table S1. Chocolate samples description.

Tag	Brand	Cocoa %	Chocolate Class	Sweetener	Fruit/Others	Expiration Date	Lot
A	Turin	73	Dark	Sugar		04/11/2017	13926
B	Lindt Excellence	70	Dark	Sugar		31/10/2016	1585
C	Turin	55	Soft dark	Sugar		03/02/2018	14560
D	Basel Chocolat	72	Dark	Acesulfame K Aspartame		30/10/2017	RJG84
E	Lindt Excellence	70	Dark	Sugar		29/02/2016	3915
F	Godiva Chocolatier	72	Dark	Sugar		25/09/2017	3261
G	Lindt Excellence	47	Soft dark	Sugar	Caramelized coconut	03/06/2016	4755
H	Lindt Excellence	47	Soft dark	Sugar	Strawberry pieces	31/08/2016	4685
I	Lindt Excellence	47	Soft dark	Sugar	Orange pieces	31/08/2016	4675
J	Lindt Excellence	47	Soft dark	Sugar	Peppermint oil	31/06/2016	4655
K	Turin	33	Milk chocolate	Sugar	Whole milk powder	21/01/2017	1610
L	Turin	NR	Dark	Maltitol		19/01/2018	13968
M	Lindt Excellence	85	Dark	Sugar		31/08/2016	3655
N	Lindt Excellence	90	Dark	Sugar		31/10/2016	3585
O	Chocolate Amatller (Ecuador)	85	Dark	Sugar		dic-17	171215
P	Chocolate Amatller (Ghana)	85	Dark	Sugar		01/12/2017	171214
Q	d'meals	NR	Soft dark	Splenda®	Strawberry pieces	04/01/2018	B64
R	MDD	NR	Soft dark	Splenda®		30/01/2018	b50
S	New Art	60	Soft dark	Sugar		22/11/2017	
T	d'meals	NR	Soft dark	Splenda®	Pistachio pieces	30/01/2018	b58
U	Turin reposteria	NR	Milk chocolate	Sugar	Whole milk powder	07/11/2017	14747
V	Chocolate Amatller	32	Milk chocolate	Sugar	Whole milk powder	01/02/2018	180208
W	Turin reposteria	NR	Soft dark	Sugar		17/01/2018	14219
X	Turin reposteria		Dark	Sugar		15/01/2018	13823
Y	Chocolate Amatller	50	Soft dark	Sugar		01/12/2017	171223
Z	Chocolate Amatller (Ghana)	32	Milk chocolate	Sugar	Whole milk powder	01/11/2017	171120

NR—Not reported in the label.

Table S2. Training parameters for both classifiers.

Training Parameter	Classifier 1		Classifier 2	
	Experiment 1	Experiment 2	Experiment 1	Experiment 2
Training protocol	Resilient backpropagation	Resilient backpropagation	Resilient backpropagation	Resilient backpropagation
Performance	Mean squared error	Mean squared error	Mean squared error	Mean squared error
Performance goal	0.0001	0.0075	0.0001	0.0001
Epoch limit	10000	10000	10000	10000
Learning rate	0.00012	0.00012	0.00012	0.00012
Minimum performance gradient	1×10^{-12}	1×10^{-12}	1×10^{-12}	1×10^{-12}

Table S3. Classifier 1, experiment 1 full test results. Accumulated classification for 10 repetitions of k-fold validation (k = 4). Bold numbers for correctly classified.

	Chocolate Samples																									
**Classified as**	**A**	**B**	**C**	**D**	**E**	**F**	**G**	**H**	**I**	**J**	**K**	**L**	**M**	**N**	**O**	**P**	**Q**	**R**	**S**	**T**	**U**	**V**	**W**	**X**	**Y**	**Z**
A	**330**	26	15	4	12	3	1	0	0	0	1	0	3	4	0	1	3	2	2	0	1	1	0	2	0	0
B	10	**329**	0	3	7	30	0	0	0	1	0	0	1	15	0	23	2	1	0	0	1	0	0	0	0	0
C	23	2	**263**	28	65	4	0	0	0	0	3	1	1	0	1	1	1	0	9	0	0	0	1	0	1	0
D	0	5	22	**315**	20	20	1	0	0	0	0	8	2	0	7	10	0	0	0	0	0	0	0	0	0	0
E	10	2	22	16	**191**	15	1	0	0	0	1	8	2	0	6	2	2	0	0	0	0	1	0	0	0	0
F	1	23	2	27	17	**182**	0	2	0	3	0	4	0	3	0	3	1	1	1	0	3	2	0	0	0	0
G	0	0	0	2	0	0	**383**	1	0	0	3	5	5	0	0	0	0	0	0	0	0	2	0	1	0	0
H	0	0	0	2	1	0	1	**389**	1	0	2	3	0	13	0	0	0	0	0	0	0	0	0	1	0	0
I	0	0	0	0	0	0	0	0	**393**	1	1	4	0	3	0	2	0	0	0	0	0	0	0	0	0	0
J	0	2	0	0	0	0	0	3	0	**390**	0	2	1	6	0	0	0	0	0	0	0	1	0	0	0	0
K	0	0	0	0	1	0	0	0	0	0	**374**	6	9	0	0	0	0	0	0	0	0	0	1	0	1	0
L	0	0	0	1	1	1	3	0	0	0	0	**342**	15	9	0	3	0	0	0	1	0	0	0	1	0	0
M	0	0	1	0	1	0	0	0	0	0	11	13	**344**	10	0	0	0	2	0	2	0	0	1	0	0	0
N	0	1	0	1	0	1	0	6	0	4	2	3	10	**363**	0	0	0	0	0	0	0	1	0	0	0	0
O	4	2	0	4	5	0	0	0	1	0	1	3	2	0	**287**	38	1	0	0	11	0	0	0	0	1	3
P	0	9	0	3	1	9	0	0	2	1	0	8	3	2	23	**301**	5	2	0	1	0	0	0	1	0	0
Q	7	3	4	0	1	1	0	0	0	0	2	2	3	7	5	7	**299**	5	2	1	0	0	0	2	0	1
R	0	3	2	0	0	4	0	0	1	5	1	0	2	12	3	7	8	**377**	0	1	1	0	0	6	0	0
S	5	0	14	1	7	7	1	0	0	0	3	2	1	1	2	0	7	1	**377**	0	1	0	0	0	0	0
T	0	0	0	0	0	1	1	0	1	0	5	10	15	0	3	0	0	0	0	**369**	0	0	0	0	4	2
U	0	0	4	2	13	2	1	0	0	0	1	0	1	0	2	0	0	0	1	0	**372**	0	6	6	0	0
V	0	0	0	3	0	4	5	1	0	0	0	1	2	3	0	1	0	0	0	0	0	**398**	0	0	0	0
W	0	0	1	0	0	1	0	0	0	0	12	0	5	0	0	0	0	0	0	5	5	0	**368**	14	2	7
X	1	0	0	2	0	2	4	1	0	5	2	2	5	8	0	2	0	0	0	0	8	0	9	**303**	1	2
Y	0	0	0	0	0	0	0	0	0	0	5	0	5	0	0	1	0	0	0	22	1	0	5	7	**363**	26
Z	0	0	1	0	1	0	2	0	0	0	1	4	1	0	13	4	0	0	0	10	0	0	3	5	12	**354**
Total analyzed	400	400	400	400	400	400	400	400	400	400	400	400	400	400	400	400	400	400	400	400	400	400	400	400	400	400

Table S4. Classifier 1, experiment 1 full training results. Accumulated classification for 10 repetitions of k-fold validation (k = 4). Bold numbers for correctly classified.

	Chocolate Samples																									
**Class.**	**A**	**B**	**C**	**D**	**E**	**F**	**G**	**H**	**I**	**J**	**K**	**L**	**M**	**N**	**O**	**P**	**Q**	**R**	**S**	**T**	**U**	**V**	**W**	**X**	**Y**	**Z**
A	**1192**	2	0	0	0	0	0	0	0	0	0	0	0	0	0	0	0	2	0	0	0	0	0	0	0	0
B	1	**1196**	0	0	1	0	0	0	0	0	0	0	0	0	0	0	0	0	0	0	0	0	0	0	0	0
C	0	0	**1126**	1	6	0	0	0	0	0	0	0	0	0	0	0	0	0	1	0	0	0	0	0	0	0
D	0	0	2	**1169**	1	0	0	0	0	0	0	0	0	0	0	0	0	0	0	0	0	0	0	0	0	0
E	0	0	0	0	**1032**	1	0	0	0	0	0	0	0	0	0	0	1	0	0	0	0	0	0	0	0	0
F	0	2	0	9	1	**1066**	0	0	0	0	0	0	0	0	0	0	0	0	0	0	0	0	0	0	0	0
G	0	0	0	0	0	0	**1199**	0	0	0	0	0	0	0	0	0	0	0	0	0	0	0	0	0	0	0
H	0	0	0	0	0	0	0	**1200**	0	0	0	0	0	0	0	0	0	0	0	0	0	0	0	0	0	0
I	0	0	0	0	0	0	0	0	**1200**	0	0	0	0	0	0	0	0	0	0	0	0	0	0	0	0	0
J	0	0	0	0	0	0	0	0	0	**1200**	0	0	0	0	0	0	0	0	0	0	0	0	0	0	0	0
K	0	0	0	0	0	0	0	0	0	0	**1197**	0	0	0	0	0	0	0	0	0	0	0	0	0	0	0
L	0	0	0	0	0	0	0	0	0	0	0	**1200**	0	0	0	0	0	0	0	0	0	0	0	0	0	0
M	0	0	0	0	0	0	0	0	0	0	0	0	**1198**	0	0	0	0	0	0	0	0	0	0	0	0	0
N	0	0	0	0	0	0	0	0	0	0	0	0	1	**1200**	0	0	0	0	0	0	0	0	0	0	0	0
O	0	0	0	0	0	0	0	0	0	0	0	0	0	0	**1154**	1	0	0	0	0	0	0	0	0	0	2
P	0	1	0	0	0	1	0	0	0	0	0	0	0	0	2	**1163**	0	0	0	0	0	0	0	0	0	0
Q	0	0	0	0	0	0	0	0	0	0	0	0	0	0	0	0	**970**	1	0	0	0	0	0	0	0	0
R	0	0	0	0	0	0	0	0	0	0	0	0	0	0	0	0	0	**1182**	0	0	0	0	0	0	0	0
S	0	0	0	0	0	0	0	0	0	0	0	0	0	0	0	0	0	0	**1173**	0	0	0	0	0	0	0
T	0	0	0	0	0	0	0	0	0	0	0	0	0	0	0	0	0	0	0	**1152**	0	0	0	0	0	0
U	0	0	0	0	0	0	0	0	0	0	0	0	0	0	0	0	0	0	0	0	**1188**	0	1	1	0	0
V	0	0	0	0	0	0	0	0	0	0	0	0	0	0	0	0	0	0	0	0	0	**1200**	0	0	0	0
W	0	0	0	0	0	0	0	0	0	0	0	0	0	0	0	0	0	0	0	1	2	0	**1187**	2	0	0
X	0	0	0	0	0	0	0	0	0	0	0	0	0	0	0	0	0	0	0	0	0	0	1	**1005**	0	0
Y	0	0	0	0	0	0	0	0	0	0	0	0	0	0	0	0	0	0	0	1	0	0	0	0	1175	6
Z	0	0	0	0	0	0	0	0	0	0	0	0	0	0	0	0	0	0	0	1	0	0	0	0	4	1168
Analyzed	1200	1200	1200	1200	1200	1200	1200	1200	1200	1200	1200	1200	1200	1200	1200	1200	1200	1200	1200	1200	1200	1200	1200	1200		

Table S5. Classifier 2, experiment 1 full test results. Accumulated classification for 10 repetitions of k-fold validation (k = 4). Bold numbers for correctly classified.

	Chocolate Samples																									
**Class**	**A**	**B**	**C**	**D**	**E**	**F**	**G**	**H**	**I**	**J**	**K**	**L**	**M**	**N**	**O**	**P**	**Q**	**R**	**S**	**T**	**U**	**V**	**W**	**X**	**Y**	**Z**
DK	**356**	**398**	119	**360**	**309**	**305**	4	2	0	1	29	62	**348**	**371**	**329**	**373**	13	9	2	12	15	10	13	**331**	4	9
SDK	39	4	**253**	31	75	65	**388**	**393**	**398**	**393**	81	**346**	59	33	56	30	**385**	**385**	**394**	**389**	21	2	**370**	28	**374**	31
ML	7	1	8	8	21	20	11	0	6	4	**301**	17	21	8	11	4	0	1	2	6	**369**	**395**	15	25	23	**352**
CO	0	0	1	1	0	0	**386**	2	1	0	5	6	3	0	0	0	0	1	0	0	0	1	0	0	0	0
ST	7	1	1	0	0	2	9	**379**	6	7	2	7	5	4	3	3	**208**	11	1	1	0	0	0	0	4	1
OR	0	0	0	0	0	0	0	0	**390**	4	3	3	1	2	0	0	0	0	0	1	0	0	0	0	0	0
MI	0	0	0	0	1	0	0	1	5	**381**	5	3	5	2	0	1	0	1	0	0	0	0	0	0	0	0
PI	0	1	0	3	2	6	2	0	0	0	18	41	8	2	0	3	1	0	0	**375**	0	0	0	4	5	2
SU	**392**	**383**	**353**	145	**364**	**300**	**398**	**389**	**385**	**391**	**358**	66	**366**	**374**	**330**	**311**	28	26	**392**	25	**397**	**399**	**397**	**395**	**391**	**398**
SP	13	10	23	25	**140**	33	0	0	1	0	6	6	12	2	26	7	0	0	1	1	3	1	2	0	0	0
Total analyzed	400	400	400	400	400	400	400	400	400	400	400	400	400	400	400	400	400	400	400	400	400	400	400	400	400	400

Table S6. Classifier 2, experiment 1 full training results. Accumulated classification for 10 repetitions of k-fold validation (k = 4). Bold numbers for correctly classified.

	Chocolate Samples																									
**Class**	**A**	**B**	**C**	**D**	**E**	**F**	**G**	**H**	**I**	**J**	**K**	**L**	**M**	**N**	**O**	**P**	**Q**	**R**	**S**	**T**	**U**	**V**	**W**	**X**	**Y**	Z
DK	**1195**	**1200**	39	**1200**	**1184**	**1191**	0	0	0	0	0	1	**1200**	**1200**	**1196**	**1200**	0	0	0	0	4	10	3	**1137**	0	0
SDK	2	0	**1160**	0	8	5	**1200**	**1200**	**1200**	**1200**	2	**1200**	0	0	5	1	**1200**	**1199**	**1200**	**1199**	15	0	**1187**	5	**1196**	8
ML	0	0	0	0	0	0	0	0	0	0	**1199**	0	0	0	0	0	0	0	0	0	**1167**	**1192**	6	8	5	**1174**
CO	0	0	0	0	0	0	**1200**	0	0	0	0	0	0	0	0	0	0	0	0	0	0	0	0	0	0	0
ST	0	0	0	0	0	0	0	**1200**	0	0	0	0	0	0	0	0	**672**	7	0	0	0	0	0	0	0	0
OR	0	0	0	0	0	0	0	0	**1196**	0	0	0	0	0	0	0	0	0	0	0	0	0	0	0	0	0
MI	0	0	0	0	0	0	0	0	0	**1200**	0	0	0	0	0	0	0	0	0	0	0	0	0	0	0	0
PI	0	0	0	0	0	0	0	0	0	0	0	0	0	0	0	0	0	0	0	**1192**	0	0	0	0	0	0
SU	**1200**	**1200**	**1195**	132	**1199**	**1195**	**1200**	**1200**	**1200**	**1200**	**1200**	0	**1200**	**1200**	**1197**	**1192**	21	30	**1195**	0	**1200**	**1200**	**1200**	**1200**	**1200**	**1200**
SP	0	0	1	1	**1021**	0	0	0	0	0	0	0	0	0	1	0	0	0	0	0	0	0	0	0	0	0
Total analyzed	1200	1200	1200	1200	1200	1200	1200	1200	1200	1200	1200	1200	1200	1200	1200	1200	1200	1200	1200	1200	1200	1200	1200	1200	1200	1200

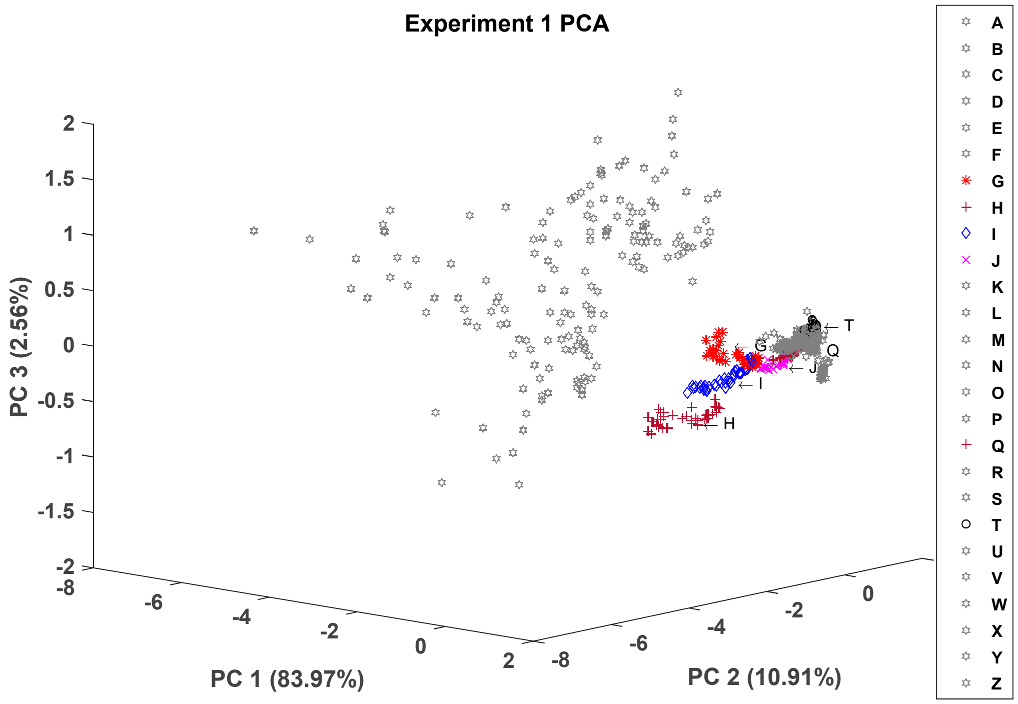

Figure S1. Experiment 1 PCA colored by extra ingredient (Gray, No extra ingredient; Red, Coconut; Wine, Strawberry; Blue, Orange; Magenta, Mint; and Black, Pistachios). A to Z tags correspond to the chocolate samples listed in Table S1.

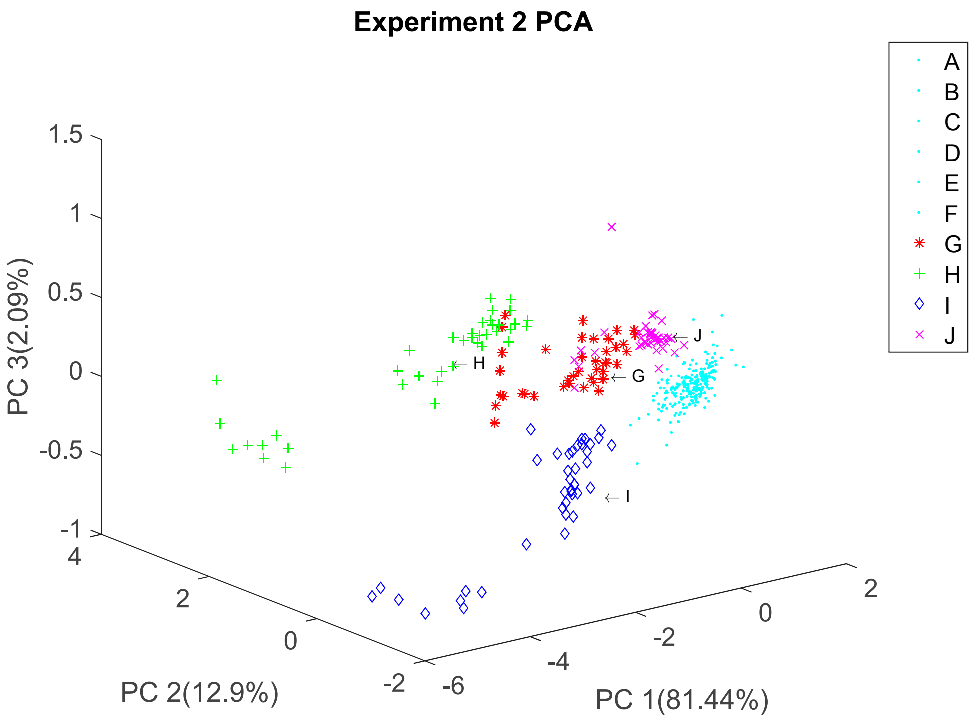

Figure S2. Experiment 2 PCA colored by extra ingredient (Cyan, No extra ingredient; Red, Coconut; Green, Strawberry; Blue, Orange; and Magenta, Mint). A to J tags correspond to the chocolate samples listed in Table S1.

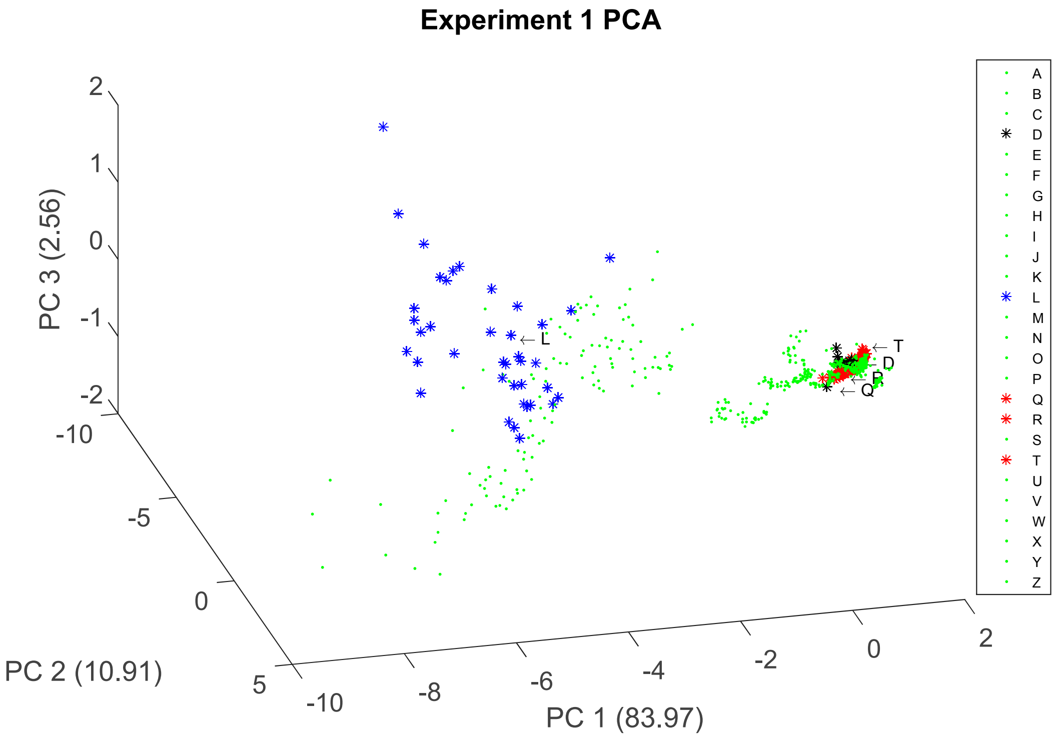

Figure S3. Experiment 1 PCA colored by Sweetener (Green, Sugar; Black, Acesulfame-K/Aspartame; Blue, Maltitol; and Red, Splenda®). A to Z tags correspond to the chocolate samples listed in Table S1.

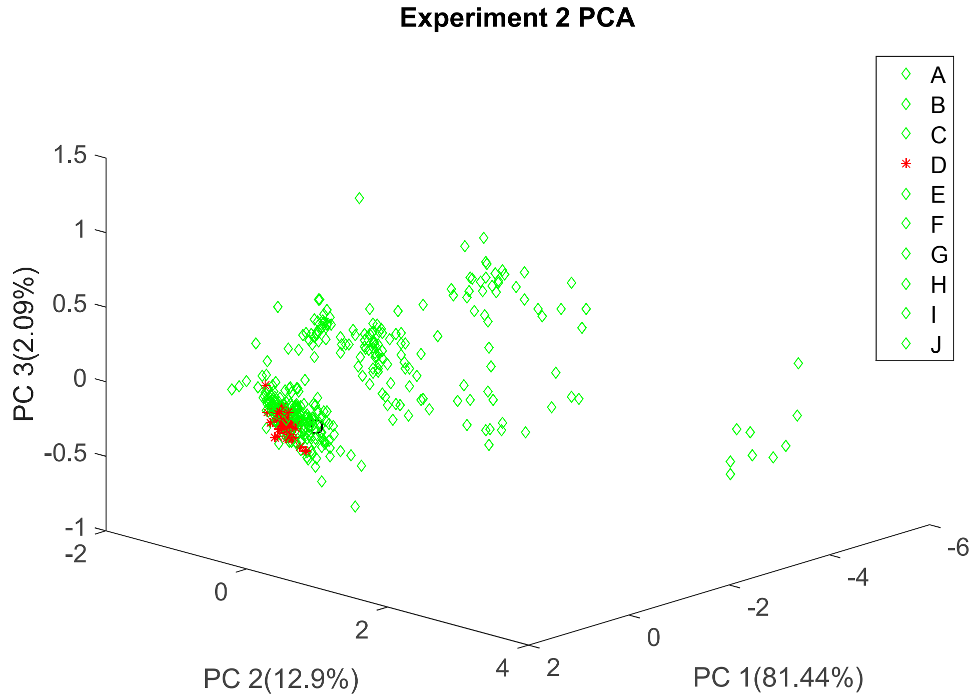

Figure S4. Experiment 2 PCA colored by Sweetener (Green, Sugar; and Red, Acesulfame-K/Aspartame). A to J tags correspond to the chocolate samples listed in Table S1.

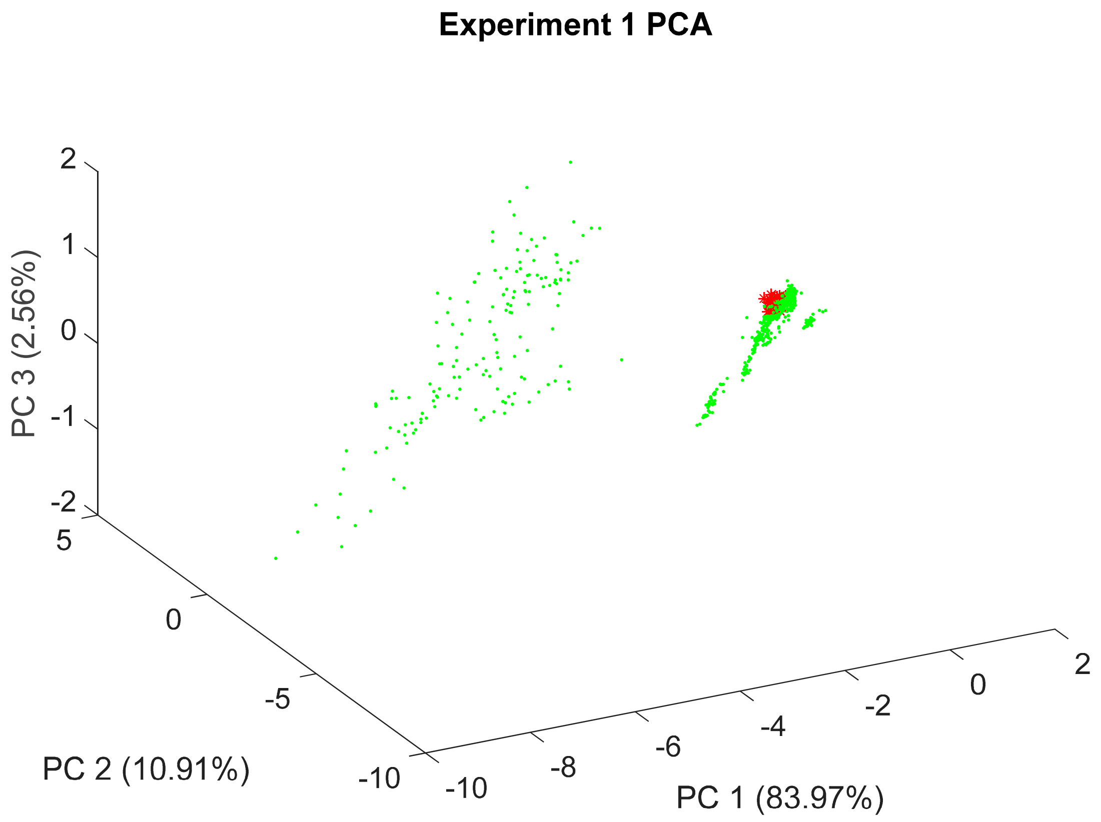

Figure S5. Experiment 1 PCA colored by expiration date (Green, Non spoiled; and Red, Spoiled).

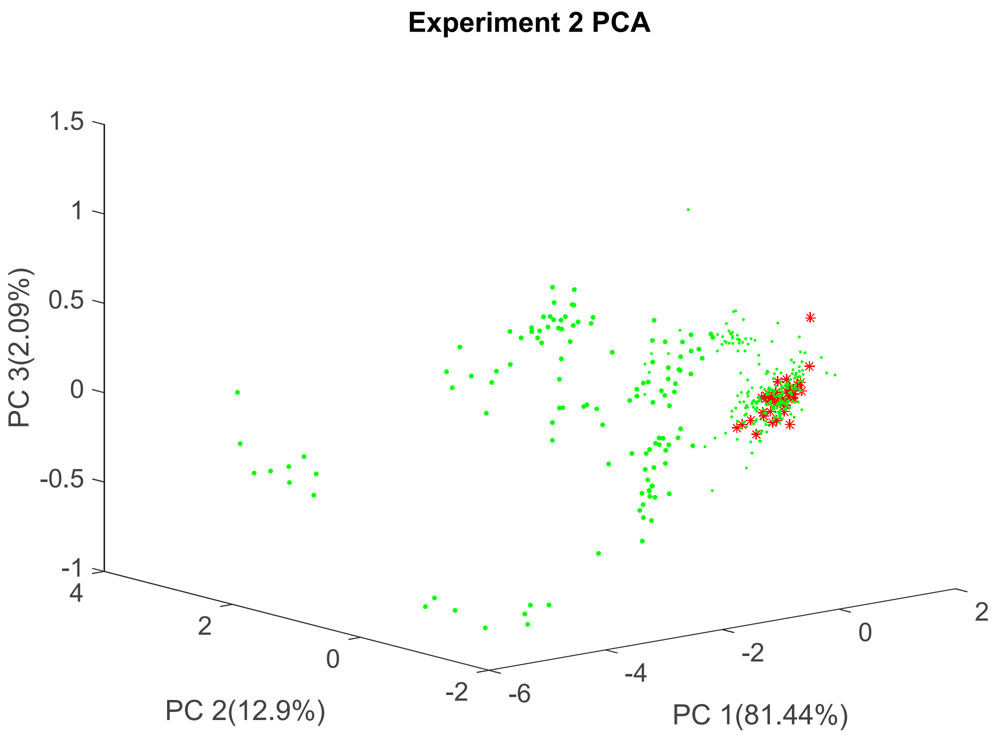

Figure S6. Experiment 2 PCA colored by expiration date (Green, Non spoiled; and Red, Spoiled).

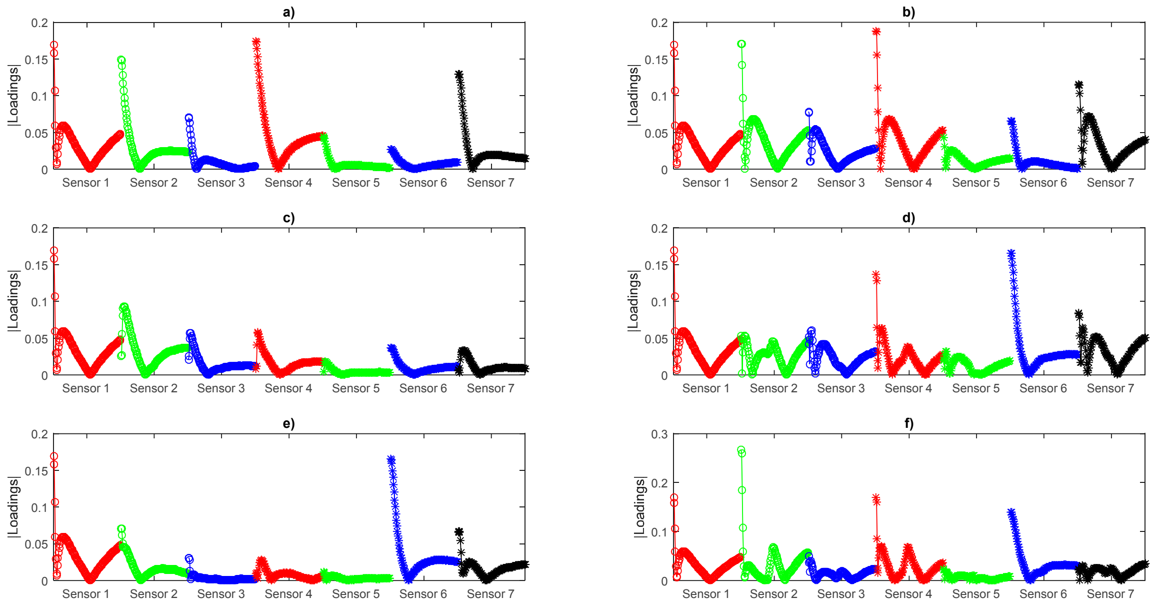

Figure S7. Experiment 1 PC1 to PC6 loadings plot (X-axis corresponding to sensor array signals and Y-axis to the absolute value of the loadings). (**a**) PC1; (**b**) PC2; (**c**) PC3; (**d**) PC4; (**e**) PC5 and (**f**) PC6.

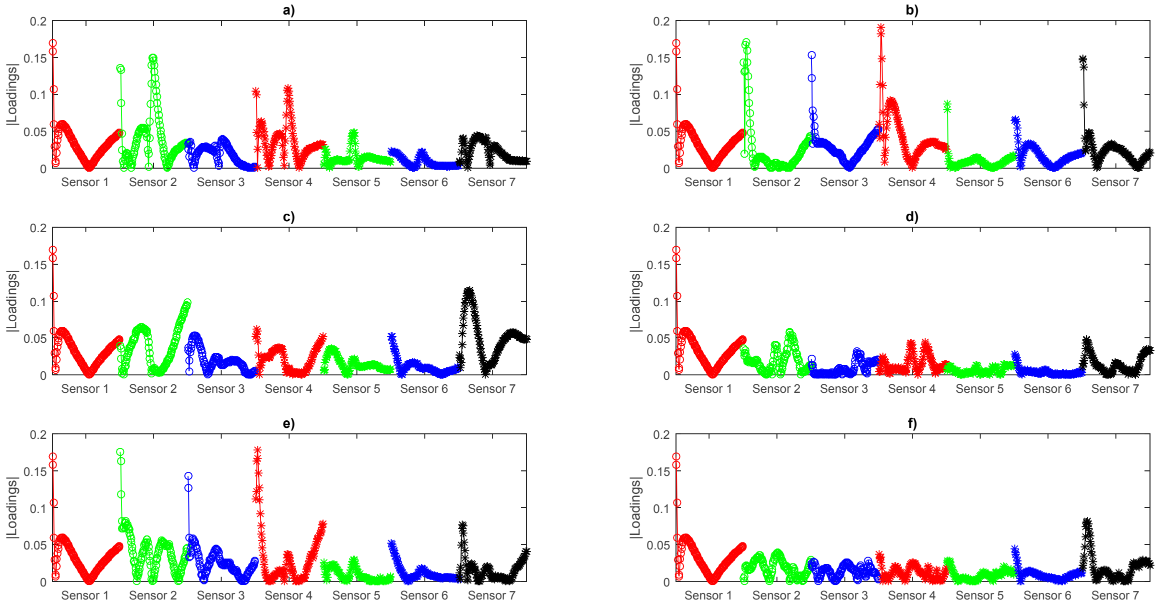

Figure S8. Experiment 1 PC7 to PC12 loadings plot (X-axis corresponding to sensor array signals and Y-axis to the absolute value of the loadings). (**a**) PC7; (**b**) PC8; (**c**) PC9; (**d**) PC10; (**e**) PC11 and (**f**) PC12.

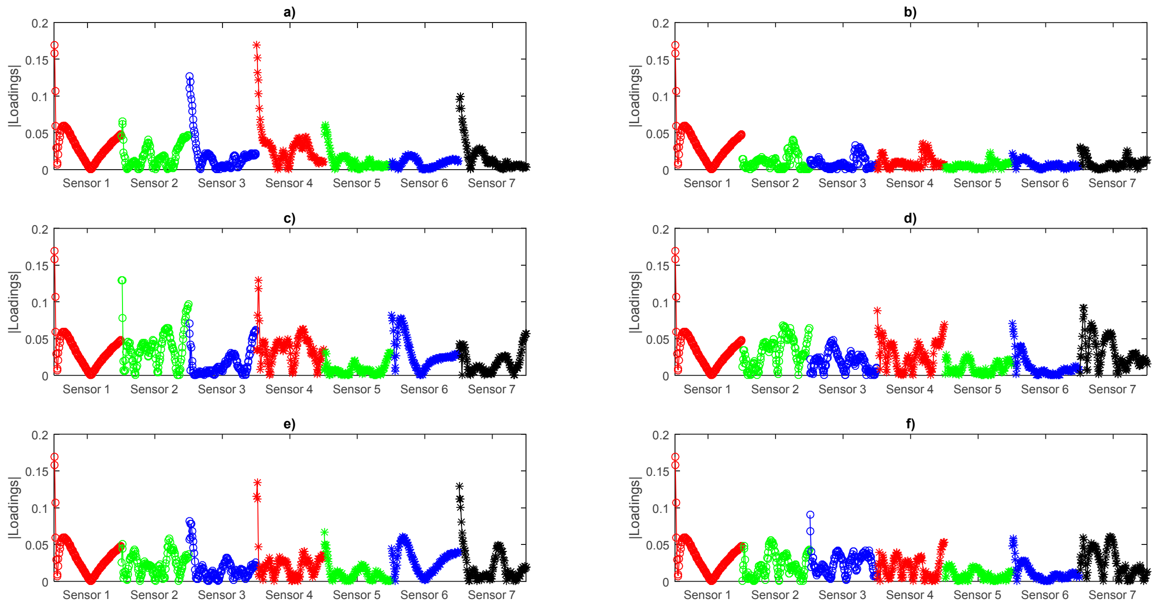

Figure S9. Experiment 1 PC13 to PC18 loadings plot (X-axis corresponding to sensor array signals and Y-axis to the absolute value of the loadings). (**a**) PC13; (**b**) PC14; (**c**) PC15; (**d**) PC16; (**e**) PC17 and (**f**) PC18.

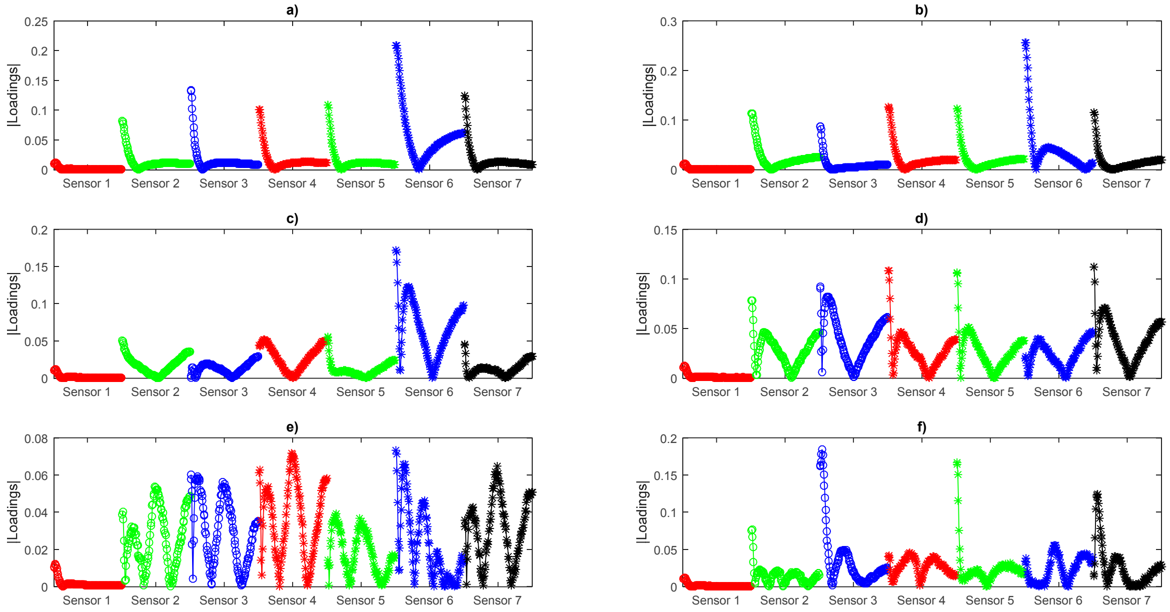

Figure S10. Experiment 2 PC1 to PC6 loadings plot (X-axis corresponding to sensor array signals and Y-axis to the absolute value of the loadings). (**a**) PC1; (**b**) PC2; (**c**) PC3; (**d**) PC4; (**e**) PC5 and (**f**) PC6.

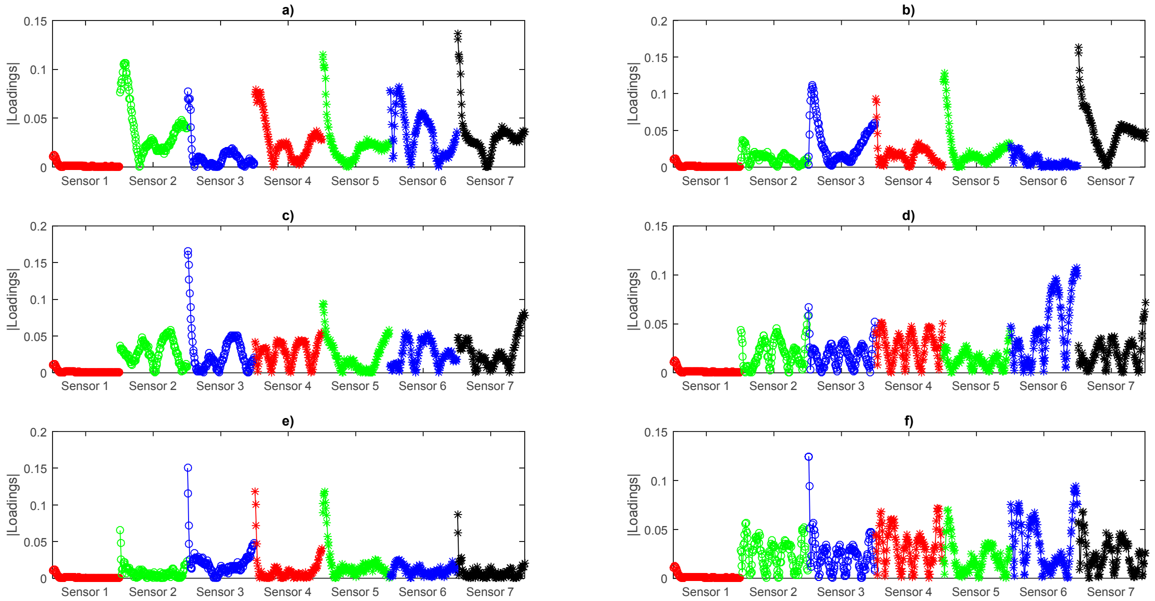

Figure S11. Experiment 2 PC7 to PC12 loadings plot (X-axis corresponding to sensor array signals and Y-axis to the absolute value of the loadings). (**a**) PC7; (**b**) PC8; (**c**) PC9; (**d**) PC10; (**e**) PC11 and (**f**) PC12.

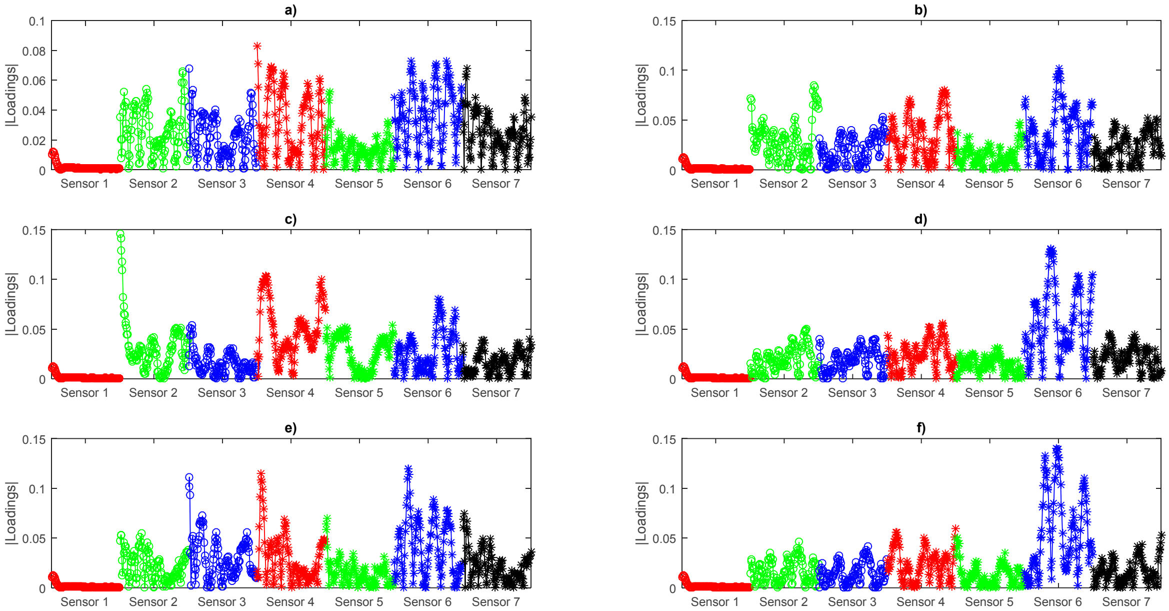

Figure S12. Experiment 2 PC13 to PC18 loadings plot (X-axis corresponding to sensor array signals and Y-axis to the absolute value of the loadings). (**a**) PC13; (**b**) PC14; (**c**) PC15; (**d**) PC16; (**e**) PC17 and (**f**) PC18.
